# Ginseng in white and red processed forms: Ginsenosides and cardiac side effects

**DOI:** 10.1002/fsn3.3879

**Published:** 2023-12-28

**Authors:** Jiexin Zhou, Jiarui Zhang, Pu Jing, Yu Lan, Xiaonian Cao, Huafang Feng, Xiaogang Liu, Qingqing Liu

**Affiliations:** ^1^ Shanghai Food Safety and Engineering Technology Research Center, Bor S. Luh Food Safety Research Center, Key Lab of Urban Agriculture Ministry of Agriculture, School of Agriculture & Biology Shanghai Jiao Tong University Shanghai China; ^2^ Luzhou Laojiao Co., Ltd. Luzhou Sichuan China

**Keywords:** Ca^2+^ signaling pathway, cardiac contractility, electrocardiography, heart rate, red ginseng and white ginseng

## Abstract

Ginseng (*Panax ginseng* Meyer) has long been consumed as a medicinal or functional food in East Asia. It is available as dried white ginseng (WG) and steamed red ginseng (RG), which might differ in ginsenoside profiles. We compared ginsenoside types of RG and WG using UPLC‐MS/MS and evaluated how they biologically affected heart of healthy rats by recording electrocardiography, measuring biochemical indicators, analyzing cardiac tissue slides, and Ca^2+^ signaling pathways. About 25 and 29 ginsenosides were detected in WG and RG, respectively, and the total ginsenoside content of RG contained was nearly 1.8 times higher than that of WG. Among them, ginsenoside Rg4, ginsenoside Rg6, ginsenoside Rh4, ginsenoside Rk1, ginsenoside Rg5, and protopanaxadiol were detected only in RG, while 20(R)‐ginsenoside Rg2 was detected only in WG. Male SD rats treated by intraperitoneal injection of WG or RG extracts were similar to the control in terms of electrocardiography and heart histology, indicating that both may not significantly affect the rats' myocardial function. However, WG and RG may induce mild cardiac injury resulting in increased cardiac collagen and creatine kinase levels. In addition, upregulated p‐CaMKII and PPARδ and downregulated SERCA2a for WG and RG treatments were further associated with increased cardiac contractility. In general, RG had less effect on the heart of healthy rats than WG, which may be due to RG having a high proportion of low‐polar ginsenosides.

## Graphical abstract

Red ginseng (RG) possessed more ginsenosides in types and abundance than white ginseng (WG). And they both showed no effect on myocardial function of healthy rats and, however, may cause mild heart damage and increase heart contractility. RG had less side effect on the heart of healthy rats than that caused by WG, possibly due to its high proportion of low‐polar ginsenosides (46%) than WG (35%).

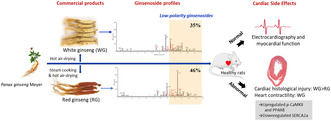

## 1 INTRODUCTION

Ginseng is a perennial plant belonging to the family of *Araliaceae*, genus *Panax* (Aminifard et al., 2021). Genus *Panax* has several species, among which *Panax ginseng* Meyer is widely cultivated in East Asia (Ratan et al., 2021). Ginseng is considered the king of herbs, so the original meaning of *Panax ginseng* Meyer was “cure‐all” (Aminifard et al., 2021). Traditional Chinese medicine has shown that ginseng is effective in enriching blood vessels, invigorating vital energy, and calming the nerves (Guo et al., 2021). Modern clinical studies also suggested the pharmacological benefits of ginseng in tumors, immunomodulation, hypertension, neurological disorders, and cardiovascular diseases, generally attributed to ginsenosides and polysaccharides as active components (Guo et al., 2021; Kim, 2018; Ratan et al., 2021).

However, ginseng's cardiac effects have long been controversial. Ginsenosides have been shown to exhibit antihypertrophic effects and may alleviate myocardial injury (Aravinthan et al., 2015; Kim & Lee, 2010; Qin et al., 2008). Conversely, ginseng extracts may cause palpitation, heart failure, diastolic dysfunction with long QT syndrome, and atrial fibrillation (Paik & Lee, 2015; Parlakpinar et al., 2019).

Processing methods of ginseng have influenced the occurrence of ginsenosides. Two typical processed products, white ginseng (WG) and red ginseng (RG), are common. WG is produced by direct sun drying or hot air‐drying processing, while RG is obtained by following the steam‐cooking and then drying processing of fresh ginseng according to Traditional Chinese Medicine (So et al., 2018). During RG processing, catabolic enzymes might be inactive, and new ginsenosides might be generated in the steam‐cooking operation by hydrolysis, dehydration, and isomerization (Nam, 2005; Xie et al., 2012). The new ginsenosides of RG were less polar and more bioavailable, which exhibited much more anticancer, antidiabetic, immunomodulatory, and neuroprotective bioactivities than their precursors (He et al., 2018; Jin et al., 2022; Nam, 2005; Xie et al., 2012).

The present study aimed to discriminate the ginsenosides of WG and RG and their effects on hearts of healthy rats, so that to provide some clues for the selection of RG and WG. We analyzed the ginsenoside profiles of WG and RG and evaluated their effects on rat heart, including electrocardiogram (ECG), related electrophysiological parameters, cardiac pathologies, and biochemical indicators. Finally, the proteins involved in Ca^2+^ signaling pathway were examined.

## 2 MATERIALS AND METHODS

### 2.1 Chemical and reagents

Lactate dehydrogenase (LDH), sarco/endoplasmic reticulum Ca^2+^‐ATPase, (SERCA), and BCA protein assay kits were supplied with Nanjing Jiancheng Bioengineering Institute (Nanjing, China), creatine kinase (CK) kit purchased from KeyGen Biotechnology Co. Ltd. (Nanjing, China), RIPA lysate buffer obtained from Solarbio Life Science (Beijing, China). Primary antibodies against calmodulin‐dependent protein kinase II (CAMKII), p‐CAMKII, SERCA2a, peroxisome proliferator activator receptor delta (PPARδ), and horseradish peroxidase (HRP)‐conjugated secondary antibody procured from Affinity Biosciences Pty Ltd. (Melbourne, Australia), and β‐Actin procured from Cell Signaling Technology (Beverly, MA, USA). Ginsenosides standards (purity≥95%) used in present work for ginseng composition analysis were purchased from Shanghai Yuanye Biotechnology Co. Ltd. (Shanghai, China). All other reagents used were of analytical grade.

### 2.2 Ginseng extract preparation and analysis of individual ginsenoside by using UPLC‐MS/MS

Red and white ginsengs dried roots (*Panax ginseng* Meyer) were purchased from Wenyuantang Pharmaceutical Co. Ltd. (Anhui, China) and placed in a brown desiccator pending use. Ginsengs were pulverized and sieved (100 mesh), and ultrasound‐assisted extraction was done by 70% ethanol (1:25, w/v) three times. The filtrate was evaporated under reduced pressure at 60°C, then lyophilized and obtained RG and WG extracts.

The UPLC‐MS/MS analysis was carried out on an Agilent UPLC‐Vion IMS Q/TOF‐MS system (Waters Corp., MA, USA) equipped with an ESI source. Individual ginsenoside separation of red and white ginseng was performed by injecting 1 μL of sample solution flow through a BEH C_18_ column (2.1 × 100 mm, 1.7 μm, Waters Corp., MA, USA) at a 0.4 mL/min flow rate. The mobile phases consisted of 0.1% formic acid–water (A) and 0.1% formic acid–acetonitrile (B), and their gradient elution program was 0–2 min, 10%–30% B; 2–6 min, 30%–70% B; 6–8 min, 70%–100% B; 8–10 min, 100% B; 10–10.1 min, 100%–10% B; 10.1–12 min, 10% B. The column oven was kept at 45°C. The mass spectrometry data in positive mode were collected range *m/z* 100–1500, and adopted parameters as follows: source temperature, 115°C; desolvation temperature, 450°C; desolvation gas flow, 900 L/h; capillary voltage, 2 kV; and collision energy, 6 eV. Identified ginsenosides were achieved by comparing their MS data with these corresponding standards or those in available references (Lee et al., 2017; Piao et al., 2020). The quantification of ginsenoside Rg1, Re, Rf, Rg2, Rb1, Rc, Rb2, and Rd were based on corresponding standard, remaining ginsenosides' equivalent concentrations quantification used ginsenoside Re as reference since not all standards were available. All data were processed with UNIFI informatics platform (Waters, Corp., vision 18.2.0).

### 2.3 Animals

Thirty male Sprague–Dawley rats weighing 300 g ± 10% were supplied by Shanghai Slack Laboratory Animal Co. Ltd. (Shanghai, China; Certificate No.: 20170005063533). All animals were housed in a standard condition at a temperature of 22 ± 2°C, humidity of 60 ± 5%, and 12 h light/dark cycle. Food and water were available ad libitum. The animals and involved experimental procedures in this study were in compliance with the regulations of Association for Assessment and Accreditation of Laboratory Animal Care International, and all protocols were approved by the Institutional Animal Care and Use Committee of Shanghai Jiao Tong University.

### 2.4 Experimental protocols

After 1 week of adaptive feeding, 30 rats were randomly allocated into three groups (10 in each): normal saline (NS) group, RG group, and WG group. Red and white ginseng extract i.p. administrated in groups RG and WG with a dose of 300 mg/kg•b.w. (Mancuso & Santangelo, 2017; Naseem et al., 2016) once a day for 28 consecutive days. The NS group rats were injected with an equal volume of normal saline. On the 1st and 28th day of experiment, the ECG of each rat was recorded under sodium pentobarbital anesthetized (3% w/v, 50 mg/kg i.p.). The normal reading is the ECG before the drug injection, the second minute after the drug injection is defined as 0 h, and two hours of ECG were traced after drug injection at an interval of half‐hour. All rats were sacrificed at the end of the ECG measurement, the blood samples were collected, heart tissues were dissected and weighed, and their ratio to body weight was cardiac hypertrophy index. Half of the heart samples were immersed in paraformaldehyde fixing solution for histological analysis, and half were stored at −80°C for further biochemical analysis.

### 2.5 Electrocardiographic analysis

Anesthetized rats were fixed on a panel in a supine position, three electrodes separately positioned into the subcutaneous tissue of right upper limb, right lower limb, and left lower limb of the rats to record the standard lead II ECG. Until the electrocardiogram on the oscilloscope was stable, the waveforms were recorded. After i.p. administration of ginseng extract or normal saline, ECGs within 2 h were collected at 0, 0.5, 1, 1.5, and 2 h for 5 min. Heart rate (HR), P‐wave, QRS‐wave, T‐wave, QRS interval, and QT interval were measured during 30 consecutive cardiac cycles to evaluate the cardiac function. The ECG traces and cardiac electrophysiological parameters analysis was performed on an RM6240 multi‐channel biological signal acquisition and processing system (Shanghai Yuyan Scientific Instrument Co. LTD., China).

### 2.6 Biochemical parameters

The activities of LDH, SERCA, and CK were measured by using commercially available kits following the instructions of manufacturer. Serum was isolated from blood by centrifugation at 1110 *g* for 15 min at 4°C. The testing tissues were taken from left ventricular tissues, homogenized, and separated the supernatant after centrifugation at 10,010 *g* for 15 min at 4°C.

### 2.7 Histological analysis

Cardiac tissues were embedded with paraffin and sectioned into 5 μm slices, and then slices were processed with hematoxylin and eosin (HE) or Masson trichrome staining to observe cardiac lesion and collagen content. Whole slide images of HE and Masson trichrome were captured with the 3DHISTECH Panoramic Scanner 250 and analyzed with CaseViewer 2.4 (3DHISTECH Ltd., Budapest, Hungary). The collagen content of heart tissues was determined by calculating the integrated optical density (IOD) by using Image Pro Plus 6.0 software.

### 2.8 Immunohistochemistry assay

Cardiac slices were incubated with 1:400 diluted PPARδ primary antibody overnight at 4°C, and then washed and incubated with secondary antibody for 1 h at room temperature. Pictures were imaged using a 3DHISTECH Panoramic Scanner 250 and analyzed with CaseViewer 2.4 (3DHISTECH Ltd., Budapest, Hungary). The relative quantification of PPARδ level in heart tissues was performed on Image Pro Plus 6.0 software by calculating the IOD.

### 2.9 Western blot analysis

The extraction of cardiac tissue protein was as the same as described in Section 2.6. BCA protein assay kit was used to measure the protein content. Then, 20ug protein was loaded on a sodium dodecyl sulfate‐polyacrylamide gel (SDS‐PAGE) and transferred to a nitrocellulose membrane. The membranes were blocked with 5% skim milk for 1 h at room temperature and incubated with primary antibodies against CAMKII (1:2000), p‐CAMKII (1:1000), SERCA2a (1:1000), PPARδ (1:1000), and β‐Actin (1:2000), respectively, overnight at 4°C. After being incubated with HRP‐conjugated secondary antibody, immunoreactive bands were visualized with enhanced chemiluminescence reagents (ECL) (Affinity Biosciences Pty Ltd., Melbourne, Australia) by a Tanon 5200 imaging system (Shanghai, China).

### 2.10 Statistical analysis

All experimental data analyses were performed by Origin 2021 software (OriginLab, Northampton, MA, USA), and the results were presented as mean (*n* = 3) ± standard error (SE). Datasets with multiple comparisons were evaluated by one‐way analysis of variance (ANOVA) followed by a Tukey's test, and the significant difference between groups was considered with *p* < .05.

## 3 RESULTS

### 3.1 UPLC‐MS/MS analysis

**FIGURE 1:**
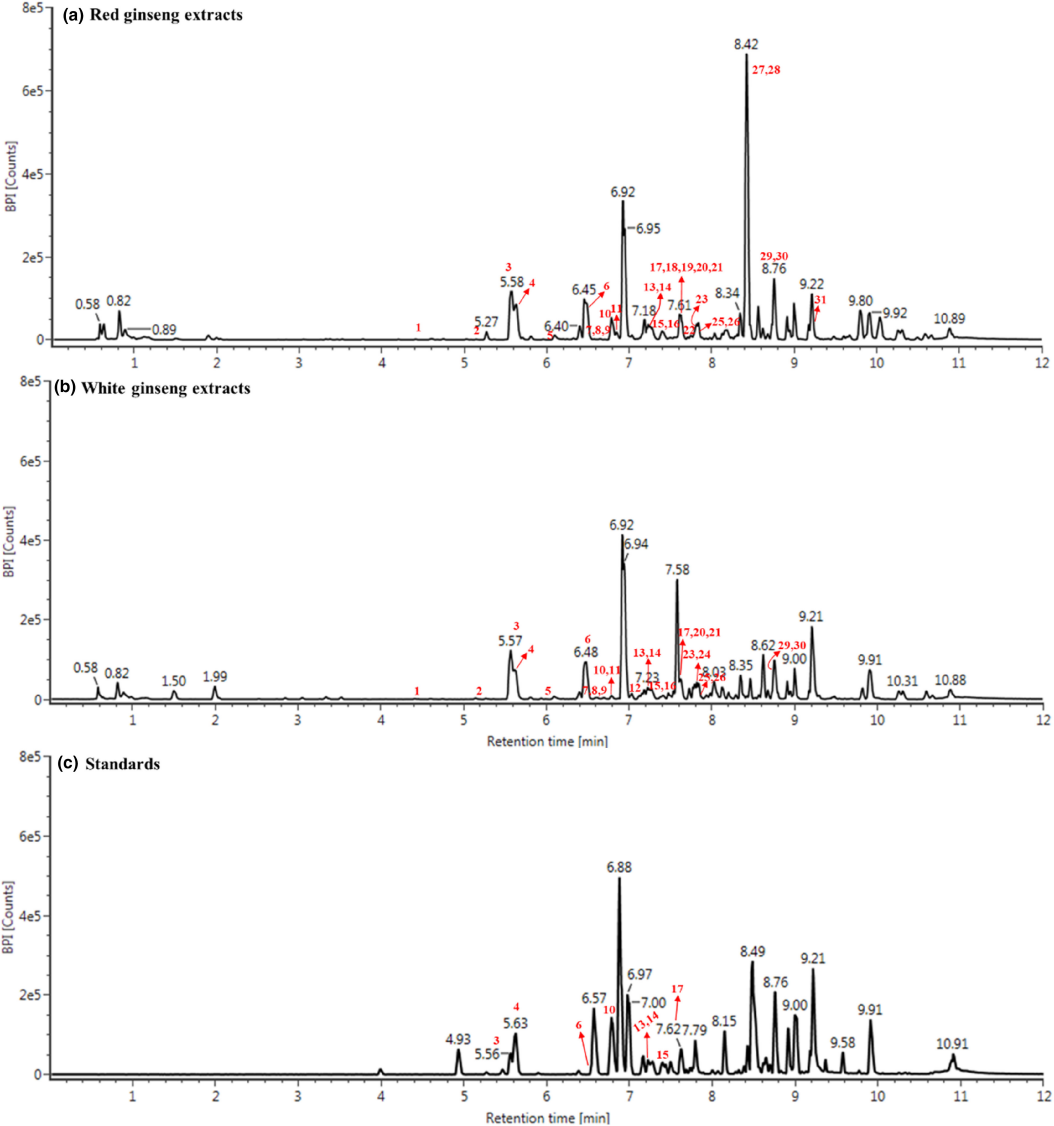
The UPLC chromatographic profiles of red ginseng extracts (a), white ginseng extracts (b), and ginsenoside standards (c).

A total of 29 and 25 major ginsenosides (Table 1) were tentatively identified and relatively quantified in RG (Figure 1a) and WG (Figure 1b), respectively, based on comparing the mass data with the standards (Figure 1c) and/or the empirical molecular formula with previous studies (Lee et al., 2017; Park et al., 2013; Wang et al., 2016; Xu et al., 2018). Several isomeric compounds were eluted simultaneously with retention times of 6.65, 7.42, 7.66, 7.79, 7.84, 8.46, and 8.72 min as shown in Table 1. Six ginsenosides were identified in RG alone, namely ginsenoside Rg4, ginsenoside Rg6, ginsenoside Rh4, ginsenoside Rk1, ginsenoside Rg5, and protopanaxadiol, while 20(R)‐ginsenoside Rg2 and ginsenoside F2 were unique to WG. And the proportion of Rk1/Rg5 in RG was high, accounting for >20% of total ginsenosides. The content of most individual ginsenosides in RG was correspondingly higher than that in WG, especially ginsenoside Rg2 and ginsenoside F3, almost 6.3‐ and 11.5‐fold of that in WG. Ginsenoside Rg1, Re, Rf, Rg2, Rd, and Rk2/Rh3 were abundant in both WG and RG. In general, the individual ginsenoside was more diverse, and the total content of ginsenosides was higher in RG than in WG. Higher ginsenoside content in RG was also found in RG than in WG (Sun et al., 2011). Those ginsenosides unique to RG might be produced due to hydrolysis, decarboxylation, and isomerization reactions, which convert a ginsenoside to anther or hydrolyze to new compounds during a steaming process of ginseng (Xu et al., 2018; Zhu et al., 2019).

**TABLE 1:** Major ginsenosides and their relative content in RG and WG detected by using UPLC‐MS/MS.

No.	Rt (min)	Compound name	Molecular formula	[M]^+^ (m/z)	Adducts	Relative content (μg/g)^a^	
						RG	WG
1	4.61	20‐*O*‐Glucoginsenoside Rf	C_48_H_82_O_19_	985.5323	+Na	18.85 ± 0.64	7.83 ± 0.31^#^
2	5.19	Notoginsenoside R1	C_47_H_80_O_18_	955.5224	+Na	4.15 ± 0.12	19.49 ± 0.62^#^
3	5.57	Ginsenoside Rg1	C_42_H_72_O_14_	823.4816	+Na	431.27 ± 20.06	450.34 ± 22.79
4	5.63	Ginsenoside Re	C_48_H_82_O_18_	969.5371	+Na	820.11 ± 37.32	652.05 ± 26.28^#^
5	6.03	Vinaginsenoside R4	C_48_H_82_O_19_	963.5504	+H	99.47 ± 4.93	59.44 ± 3.05^#^
6	6.48	Ginsenoside Rf	C_42_H_72_O_14_	823.4816	+Na	440.74 ± 23.24	234.50 ± 13.57^#^
7	6.65	Notoginsenoside R2	C_41_H_70_O_13_	753.4803	M‐H_2_O + H	9.82 ± 0.38	31.44 ± 1.08^#^
8	6.65	Ginsenoside F5	C_41_H_70_O_13_	753.4803	M‐H_2_O + H		
9	6.65	20(R)‐Notoginsenoside R2	C_41_H_70_O_13_	753.4803	M‐H_2_O + H		
10	6.78	Ginsenoside Rg2	C_42_H_72_O_13_	807.4855	+Na	557.53 ± 24.37	76.33 ± 4.36^#^
11	6.86	Ginsenoside F3	C_41_H_70_O_13_	771.4884	+H	107.14 ± 5.08	8.53 ± 0.22^#^
12	7.13	20(R)‐Ginsenoside Rg2	C_42_H_72_O_13_	785.5018	+H	Not detected	43.90 ± 1.74
13	7.24	Ginsenoside Rb1	C_54_H_92_O_23_	1131.5955	+Na	20.13 ± 0.59	113.60 ± 7.92^#^
14	7.28	Ginsenoside Rc	C_53_H_90_O_22_	1101.5852	+Na	7.28 ± 0.16	22.08 ± 0.64^#^
15	7.42	Ginsenoside Rb2	C_53_H_90_O_22_	1101.5811	+Na	11.34 ± 0.50	1.75 ± 0.04^#^
16	7.42	Ginsenoside Rb3	C_53_H_90_O_22_	1101.5811	+Na		
17	7.62	Ginsenoside Rd	C_48_H_82_O_18_	969.5375	+Na	301.91 ± 19.08	194.86 ± 9.08^#^
18	7.66	Ginsenoside Rg4	C_42_H_72_O_12_	784.5200	+NH_4_	100.22 ± 6.25	Not detected
19	7.66	Ginsenoside Rg6	C_42_H_72_O_12_	784.5200	+NH_4_		Not detected
20	7.66	Ginsenoside Rk3	C_42_H_72_O_12_	784.5200	+NH_4_		113.63 ± 6.44
21	7.66	Ginsenoside F4	C_42_H_72_O_12_	784.5200	+NH_4_		
22	7.71	Ginsenoside Rh4	C_36_H_60_O_8_	643.4171	+Na	20.56 ± 0.87	Not detected
23	7.79	20(R)/(S)‐Ginsenoside Rg3	C_42_H_72_O_13_	785.5034	+H	50.44 ± 1.34	200.65 ± 14.53^#^
24	7.79	Ginsenoside F2	C_42_H_72_O_13_	785.5034	+H	Not detected	
25	7.84	Ginsenoside Mc	C_41_H_70_O_12_	737.4845	M‐H_2_O + H	184.52 ± 7.61	190.76 ± 12.27
26	7.84	Compound Y	C_41_H_70_O_12_	737.4845	M‐H_2_O + H		
27	8.46	Ginsenoside Rk1	C_42_H_70_O_12_	789.4769	+Na	1075.58 ± 42.10	Not detected
28	8.46	Ginsenoside Rg5	C_42_H_70_O_12_	789.4769	+Na		Not detected
29	8.72	Ginsenoside Rk2	C_36_H_60_O_7_	605.4412	+H	990.29 ± 39.88	513.05 ± 37.48^#^
30	8.72	Ginsenoside Rh3	C_36_H_60_O_7_	605.4412	+H		
31	8.83	Protopanaxadiol	C_30_H_52_O_3_	483.3833	+Na	3.60 ± 0.08	Not detected
Total ginsenosides content (μg/g)						5254.95 ± 382.61	2934.23 ± 164.49^#^

*Note*: The relative content of detected ginsenosides was expressed as μg/g of dry weight and was calculated as the mean value of triplicate samples. Since not all standards were available, it cannot distinguish these ginsenosides at the same retention time, so ginsenosides with the same retention time were quantified once. The superscript “#” on WG relative content means significant differences between RG under the same retention time (*p* < .05).

^a^ The contents of Ginsenoside Rg1, Re, Rf, Rg2, Rb1, Rc, Rb2, and Rd were quantified based on corresponding standard; the relative quantification of remaining ginsenosides used ginsenoside Re as reference.

^#^ Represented a significant difference in the relative content of ginsenosides between RG and WG at the same retention time (*p* < .05).

### 3.2 Cardiac waveforms and ECG parameters

**FIGURE 2:**
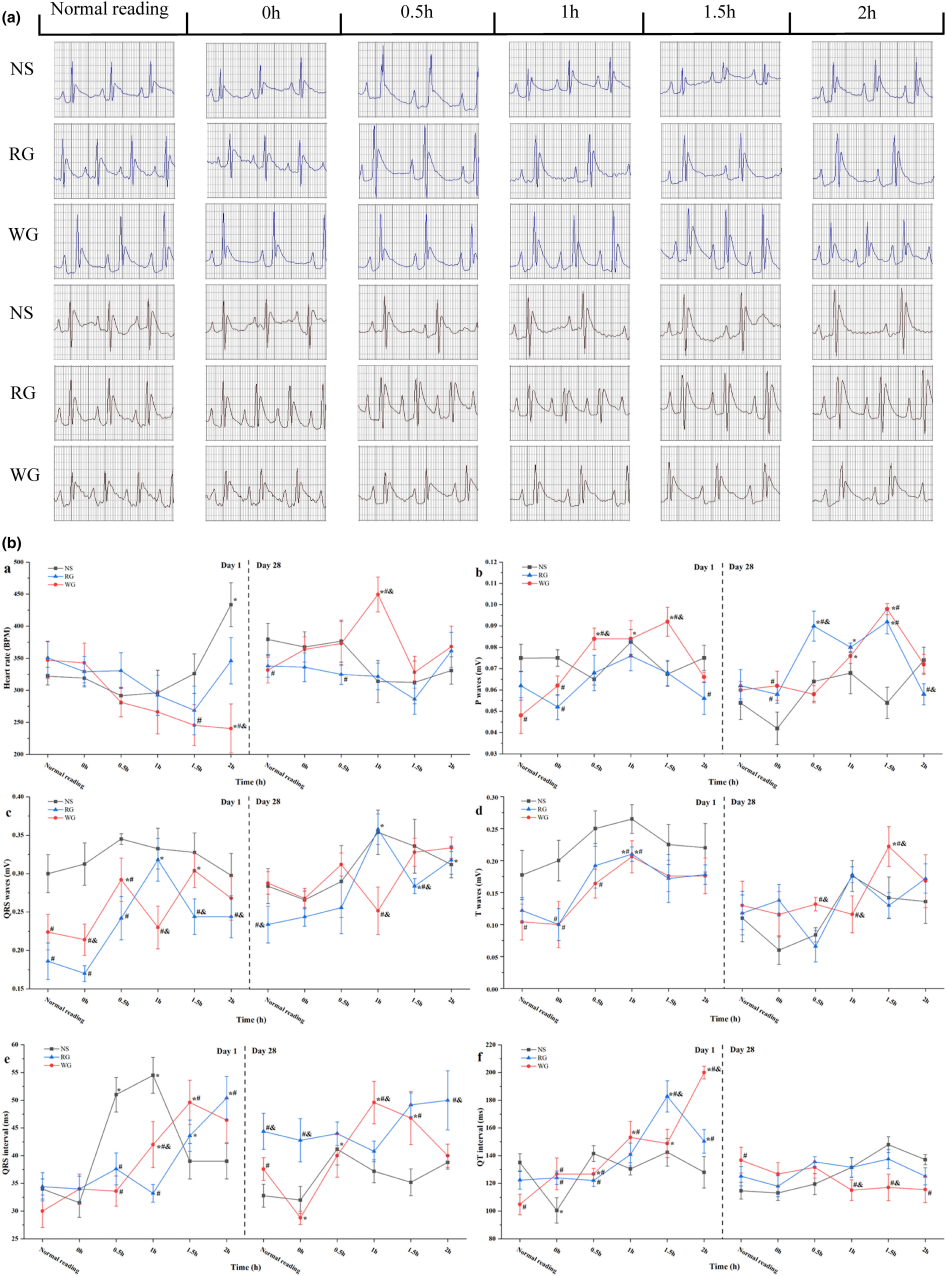
Cardiac waveforms and ECG parameters of rats within 2 h after normal saline/RG/WG i.p. administration. a, 2‐h‐ECG profiles of rats on day 1 (blue trace) and 28 (brown trace) after drug injection. b, the ECG parameters changes within 2 h, heart rate (a), p‐waves (b), QRS waves (c), t‐waves (d), QRS intervals (e), and QT intervals (f) were obtained by measuring 30 consecutive cardiac cycles at each collection site. RG: red ginseng; WG: white ginseng; NS: normal saline; ECG: electrocardiography. “*” was a symbol of significant differences with normal reading in the same group (*p* < .05); “#” represented significant differences with NS group at the same time point (*p* < .05); “&” signified significant differences between RG and WG groups (*p* < .05).

An electrocardiogram (ECG) is a recording of the electrical currents that flow through the heart as a result of the excitation–contraction coupling of heart muscle (Ho et al., 2011), and we recorded the 2 h of ECG of rats on days 1 and 28 after ginseng extract administration with blue and brown traces (Figure 2a), respectively. ECG allows the examination and visualization of heart conditions based on characteristic waves and intervals (Hadjem et al., 2014; Ho et al., 2011). According to the ECG traces in Figure 2b(a), ginseng treatment did not cause any HR difference with the NS group at the first 1 h, and the WG group showed significantly lower HR values than the NS group at 1.5 h on day 1 (*p* < .05). Their average HR within 2 h was 331 ± 52 BPM (NS group), 320 ± 47 BPM (RG group), and 287 ± 32 BPM (WG group) separately. The average HR between the RG and NS groups was not significantly different (*p* > .05) but significantly higher than that of the WG group (*p* < .05). Their corresponding average values on day 28 were 347 ± 31 BPM, 328 ± 24 BPM, and 369 ± 44 BPM, respectively, which were higher than on day 1. However, they were not significantly different from each other (*p* > .05).

The P, QRS, and t‐waves within 2 h were fluctuating in Figure 2b(b–d), but no significant differences were observed among the three groups on day 1 in terms of their mean amplitudes. After 28 days of intraperitoneal injection, red and white ginseng made the average amplitudes of p‐ and t‐waves slightly larger than the NS group: p‐wave, 0.06 ± 0.01 mV (NS group) versus 0.07 ± 0.01 mV (RG group) versus 0.07 ± 0.01 mV (WG group); t‐wave, 0.12 ± 0.04 mV (NS group) versus 0.13 ± 0.01 mV (RG group) versus 0.15 ± 0.04 mV (WG group); QRS, 0.31 ± 0.03 mV (NS group) versus 0.28 ± 0.05 mV (RG group) vs. 0.30 ± 0.03 mV (WG group). In terms of QRS and QT intervals (Figure 2b(e–f)), the average QRS intervals of RG and WG groups were shorter, and the average QT intervals of them were longer compared with the NS group on day 1. However, no significant changes were observed among the three groups (*p* > .05). Ginseng administration for 28 days shortened the average QT interval and extended average QRS intervals than day 1. Compared with the NS group, WG prolonged QRS interval but shortened QT interval, while the RG group prolonged both intervals. In general, no abnormality in ECG was observed in the three groups during a 2 h recording on 1st and 28th day.

### 3.3 Cardiac biochemical indexes

**FIGURE 3:**
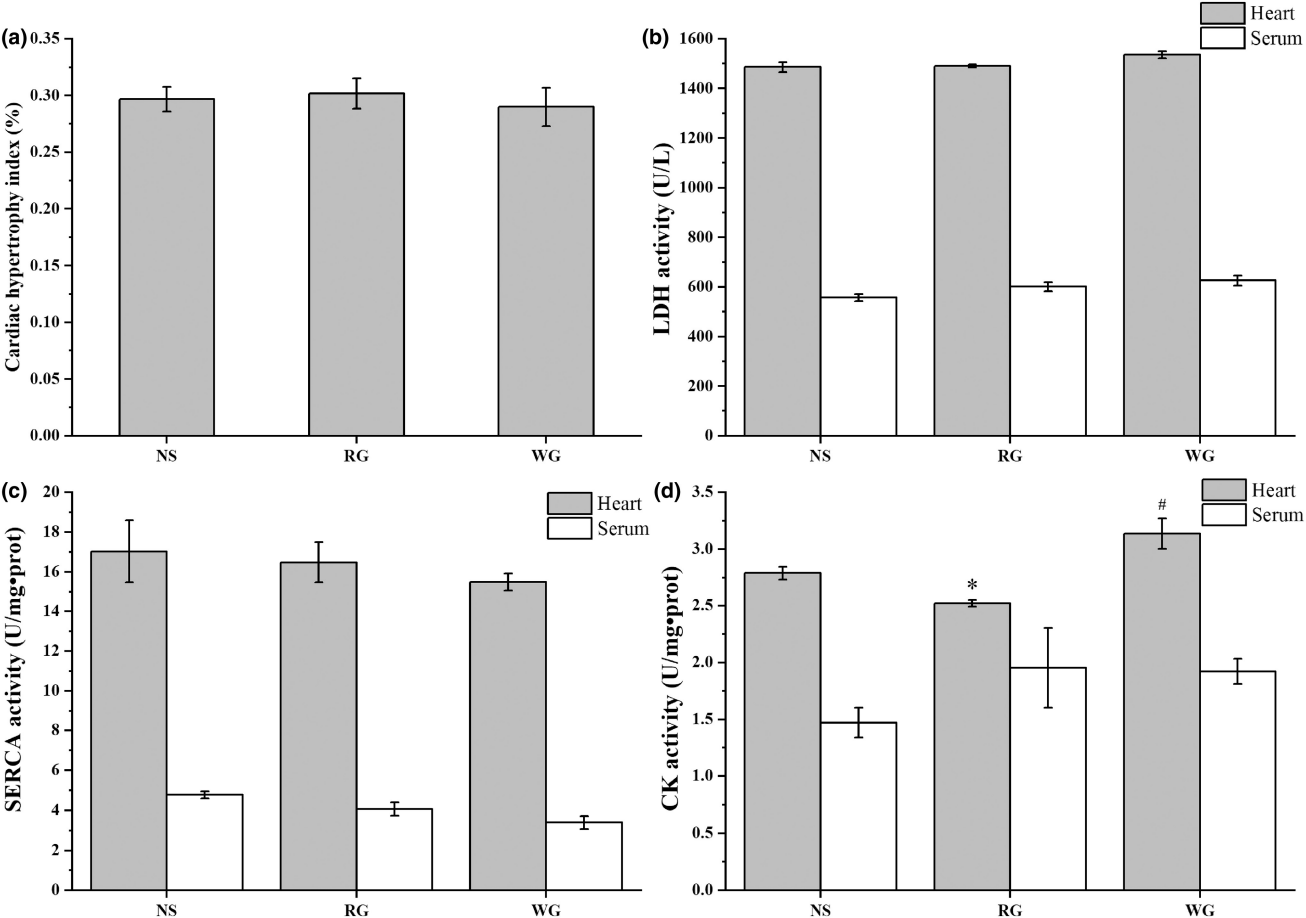
Effects of RG and WG on cardiac hypertrophy index (a), and activities of LDH (b), SERCA (c), and CK(d) in the serum and heart of rats. RG: red ginseng; WG: white ginseng; NS: normal saline; LDH: lactic dehydrogenase; SERCA: sarco/endoplasmic reticulum Ca^2+^‐ATPase; CK: creatine kinase. *p < .05 vs. NS; #*p* < .05 vs. RG.

The cardiac hypertrophy indexes were 0.29 and 0.30, respectively, for WG and RG groups (Figure 3a), which were not significantly different from the NS group (*p* > .05). LDH, SERCA, and CK activities in serum and heart were presented in Figure 3b–d. No significant differences in LDH in serum and heart were observed among three groups (*p* > .05) with heart LDH around 1500 U/L and serum LDH around 600 U/L (Figure 3b), which were consistent with previous reports (Dizaji et al., 2021; Sun et al., 2014). Both RG and WG administrations slightly decreased SERCA activity in heart and serum. The variation in the WG group was greater, and the decrease was 11% and 29% in heart and serum, respectively, in contrast to the NS group (Figure 3c). As for CK (Figure 3d), the activity in serum has a slight increase in RG and WG groups, and the activity in the heart of the WG group was about 1.12 times that of the NS group (*p* < .05).

### 3.4 Histopathological and immunohistochemical analyses

**FIGURE 4:**
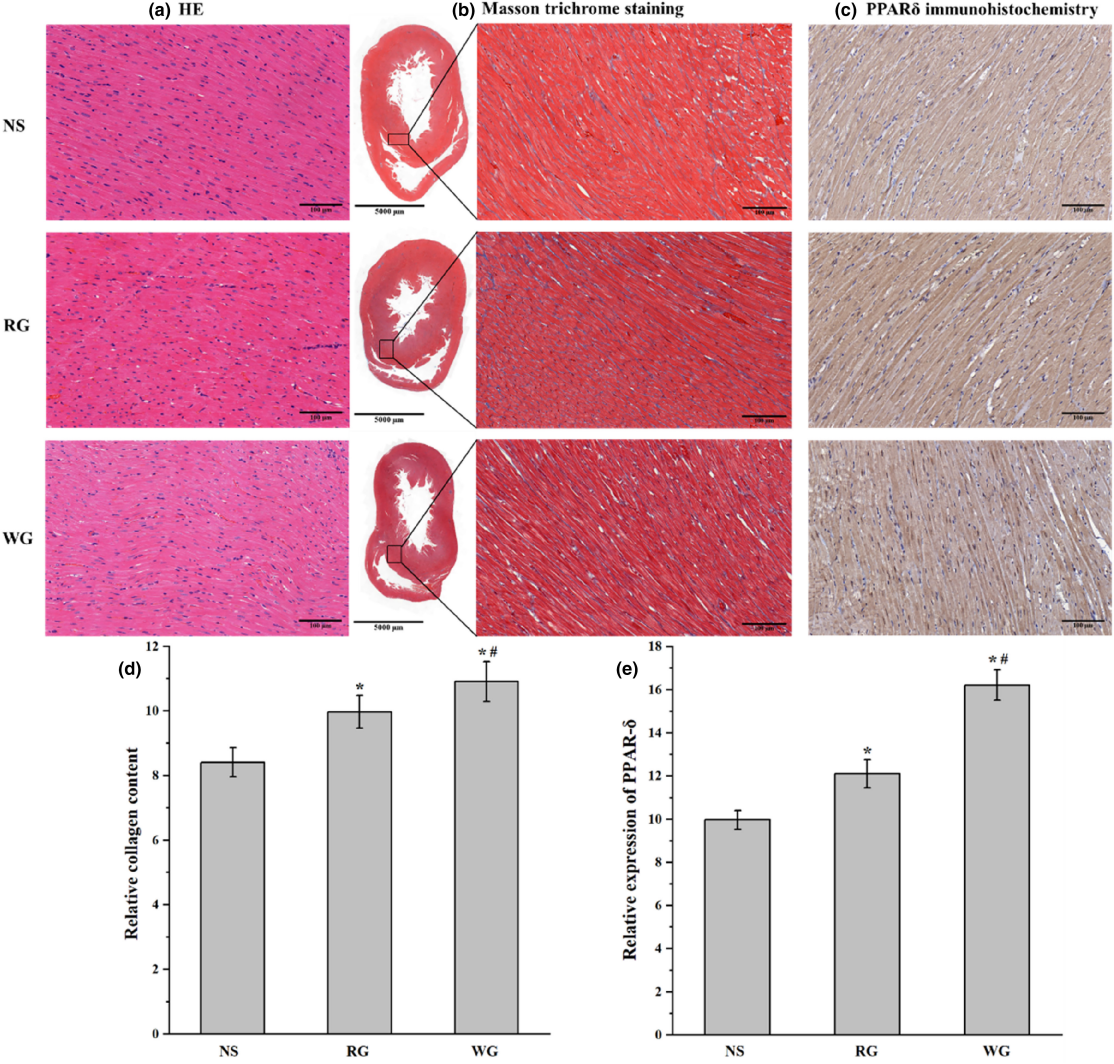
Histopathological evaluation and immunohistochemistry of cardiac tissues. a, HE staining, ×100. b and d, Masson trichrome staining (b) and relative collagen content in cardiac tissues (d); the full view of hearts in B with a magnification of ×2, and the right box areas at a higher magnification of ×100. c and e, immunohistochemistry of PPARδ (c) and its relative expression in cardiac tissues (e); c with magnification of ×100. RG: red ginseng; WG: white ginseng; NS: normal saline; HE: Hematoxylin and Eosin; PPARδ: peroxisome proliferator activator receptor delta.

As shown by HE staining, the cardiac tissues of the three groups had a normal histological appearance, with myocardial fibers arranged regularly and nuclei distributed centrally (Figure 4a). Red and white ginseng treatment did not cause any significant changes in cardiac histopathology. Masson trichrome staining gradually darkened from the NS group to the RG group to the WG group, which might be more blue staining in myocardial fiber of WG and RG groups (Figure 4b). These blue dyes deposited collagen, which was an indication of damage and fibrosis (Farag et al., 2021). The IOD quantified results manifested that the collagen contents in RG and WG groups were 1.19 and 1.30 times that of the NS group, respectively, in Figure 4d (*p* < .05). The results of PPARδ immunohistochemistry (Figure 4c) were similar to the Masson trichrome staining. Ginseng administration significantly increased the PPARδ level (*p* < .05). The levels in RG and WG groups were 1.21 times and 1.63 times that of the NS group, respectively (Figure 4e).

### 3.5 Ca^2+^ signaling pathway

**FIGURE 5:**
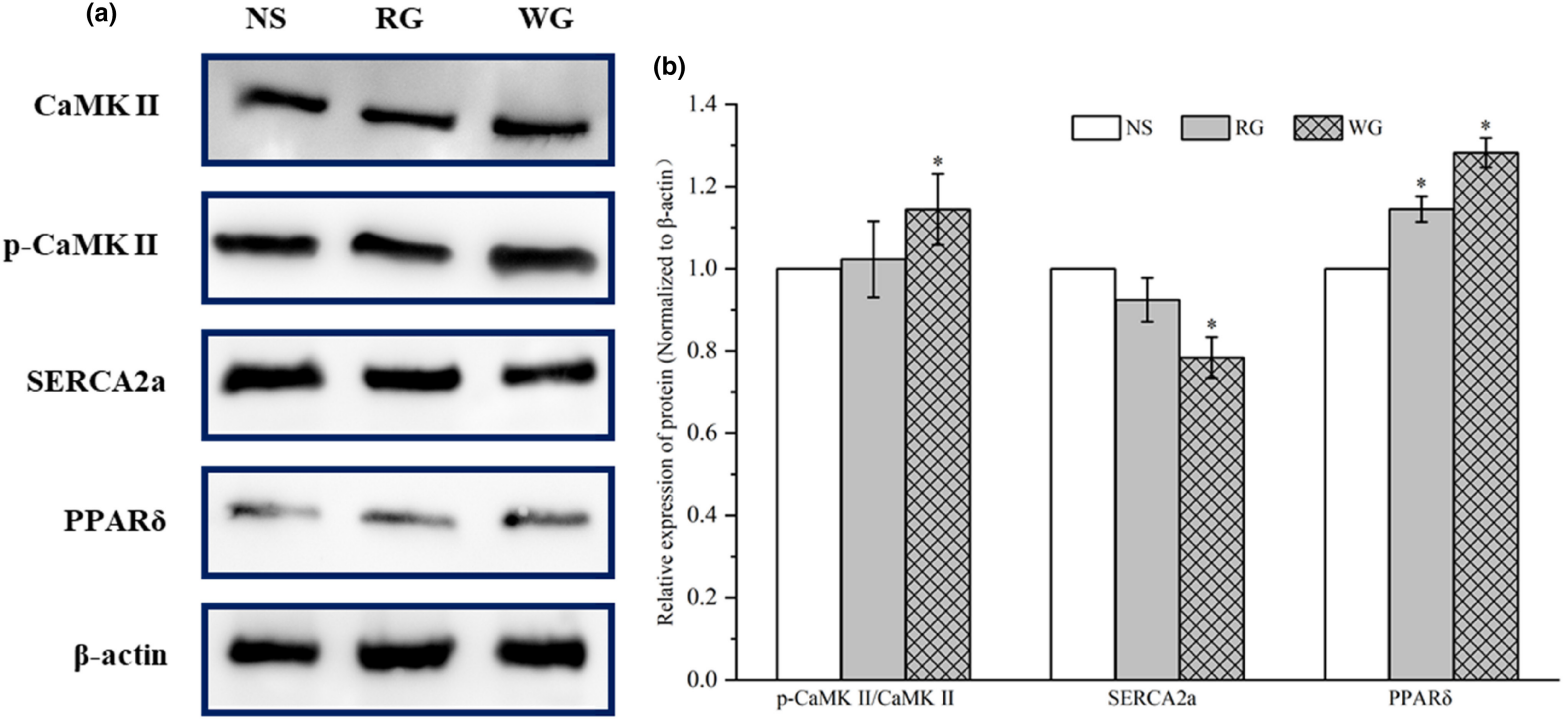
Effect of RG and WG on the expressions of proteins involved in Ca^2+^ signaling pathway. a, the immunoblot of CaMKII, SERCA2a, and PPARδ; b, the relative expressions of these proteins by comparison with the NS group according to the grayscale. RG: red ginseng; WG: white ginseng; NS: normal saline; CaMKII: calmodulin‐dependent protein kinase II; SERCA 2a: sarco/endoplasmic reticulum Ca^2+^‐ATPase 2a; PPARδ: peroxisome proliferator activator receptor delta. *p < .05 vs. NS.

The levels of proteins involved in the Ca^2+^ signaling pathway, including CaMKII, SERCA2a, and PPARδ, were detected by Western blot (Figure 5a). The WG and RG increased the phosphorylation of CaMKII, the expression level of which in the WG group was approximately 1.14 times that of the NS group (*p* < .05) (Figure 5b). Ginseng treatment also upregulated PPARδ, which was expressed by 1.28‐fold (WG) and1.15‐fold (RG) of that in the NS group (*p* < .05). SERCA2a level was downregulated in both treatment groups, which decreased by 8% in the RG group and 22% in the WG group compared with the NS group (*p* < .05).

## 4 DISCUSSION

Heart is the engine of the body, which transports nutrients and energy to the whole body by pumping blood around the body with constant ECC (Hoang‐Trong et al., 2015). Normal physiological structure and function of heart is a prerequisite for cardiac activity. HE staining and cardiac hypertrophy indexes showed normal heart histology under ginseng administration, ECG recordings and LDH levels also did not observe any abnormalities compared with the NS group. These suggested that both RG and WG may have no effect on physiological structure and function of the myocardium of healthy rats.

Other cardiac biochemical indexes are CK/SERCA, an enzyme localized in myocytes and leakage during myocardial injury, or an index of cardiac myocyte dysfunction (Chen et al., 2018; Zhang et al., 2016). CK activity increased and SERCA activity slightly decreased in ginseng‐treated groups. This suggests that the hearts in the treatment groups might suffer from myocardial injury and dysfunction to such a minor degree that it is barely detectable by ECG. On closer inspection of the results, RG administration only caused an increase in serum CK activity, and a lesser decrease in SERCA activity was also observed in the RG group (Figure 3c). These suggest that WG may cause a greater impact on heart than RG did. The degree of collagen deposition in RG and WG groups, as shown by Masson staining, was also in support of this speculation.

In addition, SERCA is responsible for pumping Ca^2+^ back into the sarcoplasmic reticulum during diastole (Eisner et al., 2017). Accordingly, CaMKII regulates the Ca^2+^ influx by controlling the switching of L‐type Ca^2+^ channels during systole (de Souza et al., 2019; Maier, 2005). The disruption of cytoplasmic calcium concentration ([Ca^2+^]_i_) homeostasis will contribute to arrhythmia (Zhang et al., 2016). In this study, overexpressed p‐CaMKII, downregulated SERCA2a, and lowered SERCA activity in ginseng‐treated groups, suggesting an increase in intracellular [Ca^2+^]_i_. This indicated WG and RG administration may induce arrhythmia. Moreover, activation of CaMKII could improve cardiac contractility (Kaurstad et al., 2012). The expression and activity reduction of SERCA2a also committed to the improvement of myocardial contractility (Zhang et al., 2016). Thus, WG and RG might also have cardiac contractility‐enhancing effect. The upregulated PPARδ also confirmed this conclusion. PPARδ expression is calcium‐ and calmodulin‐dependent, which is also a protein highly associated with cardiac contractility (Chou et al., 2012; Lin et al., 2014).

In general, WG may have a slightly greater impact than RG in terms of cardiac injury and protein expression. Normally, ginseng with higher ginsenoside content tends to exhibit greater biological activity (Ma et al., 2017; Yun et al., 2004). In a research on antiproliferative and pro‐apoptotic effects of ginseng on human colorectal cancer cells, RG (*Panax ginseng* and *Panax quinquefolius*) with higher ginsenoside content than corresponding WG exhibited stronger bioactivity (Sun et al., 2011). RG, richer in individual ginsenosides and total content than WG's, showed less impact on heart of healthy rats in present study, one reason may be due to the reduced polarity of ginsenoside profile in RG, such as ginsenoside F2, Rg6, Rh4, Rh3, Rk1, Rg5, and protopanaxatriol (Chen et al., 2020; Xie et al., 2012), together in a high percentage (>46%). Many studies have reported that the steaming and heating process could transform the polar ginsenoside of ginseng to less polar ginsenoside, endowing RG with higher medical safety and more pharmacological benefits, such as lower neurotoxicity and stronger anticancer activity and immune activity than unprocessed ginseng (Shin et al., 2018). Other constitutions' changes in processing, like polysaccharides and amino acids, may also be related to the heart impact differences; our future work will further elucidate whether these heart impacts are induced by ginsenosides or synergistically together with others in ginseng.

## 5 CONCLUSIONS

RG and WG administration had no influence on the physiological structure and function of rats' myocardium, while it could induce arrhythmia and strengthen cardiac contractility, which could be associated with upregulated p‐CaMKII and PPARδ, downregulated SERCA2a and elevated intracellular [Ca^2+^]_i_. Moreover, RG and WG would cause a slight injury to the heart. RG caused the improvement of cardiac contractility, and the degree of injury was less than that caused by WG, which might be attributed to the high proportion of low‐polarity ginsenosides and more moderate pharmacological effects of RG.

## AUTHOR CONTRIBUTIONS

**Jiexin Zhou:** Investigation (lead); methodology (lead); writing – original draft (lead). **Jiarui Zhang:** Formal analysis (equal); investigation (equal); methodology (equal). **Pu Jing:** Funding acquisition (lead); project administration (lead); supervision (lead); writing – review and editing (lead). **Yu Lan:** Resources (lead). **Xiaonian Cao:** Resources (lead). **Huafang Feng:** Investigation (equal); methodology (equal). **xiaogang Liu:** Investigation (equal); methodology (equal). **qingqing Liu:** Investigation (equal); methodology (equal).

## FUNDING INFORMATION

This work was financially supported by the Luzhou Laojiao Co. Ltd. (NO. 21H010101946).

## CONFLICT OF INTEREST STATEMENT

The authors declare that they have no conflict of interest.

## ETHICS STATEMENT

The animals and involved experimental procedures in this study were in compliance with the regulations of Association for Assessment and Accreditation of Laboratory Animal Care International, and all protocols were approved by the Institutional Animal Care and Use Committee of Shanghai Jiao Tong University.

## Data Availability

The data that support the findings of this study are available on request from the corresponding author.
